# Housekeeping gene stability in *Pesudomonas aeruginosa* PAO1 under the pressure of commonly used antibiotics in molecular microbiology assays

**DOI:** 10.3389/fmicb.2023.1140515

**Published:** 2023-03-13

**Authors:** Lingning Meng, Xiaoli Cao, Chuchu Li, Jia Li, Hui Xie, Jiping Shi, Mei Han, Han Shen, Chang Liu

**Affiliations:** ^1^Department of Laboratory Medicine, Nanjing Drum Tower Hospital, The Affiliated Hospital of Nanjing University Medical School, Nanjing, Jiangsu, China; ^2^Department of Acute Infectious Disease Control and Prevention, Jiangsu Provincial Center for Disease Control and Prevention, Nanjing, China

**Keywords:** housekeeping gene, *Pseudomonas aeruginosa*, qRT-PCR, BestKeeper, geNorm, NormFinder, RefFinder

## Abstract

*Pseudomonas aeruginosa* is an opportunistic human pathogen notorious for its remarkable capacity of multi-drug resistance, and has become one of the most important model bacteria in clinical bacteriology research. Quantitative real-time PCR is a reliable method widely used in gene expression analysis, for which the selection of a set of appropriate housekeeping genes is a key prerequisite for the accuracy of the results. However, it is easy to overlook that the expression level of housekeeping gene may vary in different conditions, especially in the condition of molecular microbiology assays, where tested strains are generally cultured under the pre-set antibiotic selection pressures, and how this affects the stability of commonly used housekeeping genes remains unclear. In this study, the expression stability of ten classic housekeeping genes (*algD, gyrA, anr, nadB, recA, fabD, proC, ampC, rpoS,* and *rpsL*) under the pressure of eight laboratory commonly used antibiotics (kanamycin, gentamycin, tetracycline, chloramphenicol, hygromycin B, apramycin, tellurite, and zeocin) were tested. Results showed that the stability of housekeeping gene expression was indeed affected by the types of antibiotics added, and of course the best reference gene set varied for different antibiotics. This study provides a comprehensive summary of the effects of laboratory antibiotics on the stability of housekeeping genes in *P. aeruginosa*, highlighting the necessity to select housekeeping genes according to the type of antibiotics used in the initial stage of experiment.

## Introduction

*Pseudomonas aeruginosa* is a gram-negative, rod-shaped bacterium belonging to the *Pseudomonadaceae* family. It is a ubiquitous organism found in many environments including soil, water, animals and humans (Moradali et al., 2017). *Pseudomonas aeruginosa* is seldom a member of normal microbial flora in most healthy adults, but a severe menace to immunocompromised patients, associated with an array of life-threatening infections, including cystic fibrosis, ventilator-associated pneumonia, urinary tract infections, burn injury infections, and bloodstream infections (Malhotra et al., 2019).

In bacteriology research, *P. aeruginosa* serves as a model organism for studying biofilm formation, type VI secretion system (T6SS) and quorum sensing signaling, etc. (Lee and Zhang, 2015; Pena et al., 2019; Thi et al., 2020). Clinically, *P. aeruginosa* is notorious for its complicated antimicrobial resistance mechanisms, which can develop antibacterial resistance either exogenously by obtaining various resistance gene cassettes, or endogenously by altering the expression level of chromosomally encoded enzymes (Breidenstein et al., 2011). Both strategies would severely limit therapeutic options and make the pathogen difficult to eradicate in clinical anti-infection treatments (Botelho et al., 2019). For these reasons, numerous studies that aim to reveal the deep patterns of *P. aeruginosa* have been performed using expressed sequences tags (ESTs), luciferase reporter system, and quantitative real-time PCR (Bustin and Mueller, 2005; Parkinson and Blaxter, 2009; England et al., 2016).

The quantitative real-time PCR is a reliable technique that is used extensively for gene expression analysis due to its high sensitivity and repeatability (Bustin and Mueller, 2005). This method could be performed as either an absolute measurement using a pre-prepared external standard curve, or as a relative measurement by comparing the expression level of a target gene with that of a presumed constantly expressed “housekeeping gene.” The expression level of housekeeping genes may be affected by a variety of factors, and therefore a selection process with caution is required (Kozera and Rapacz, 2013). For instance, in most molecular microbiology assays, tested strains which carry pre-introduced antimicrobial resistance cassette are firstly plated on the selective agar medium for colonies formation, and cultured under the same antimicrobial pressure during subsequent experimental process. Because of the great differences in pharmacological properties among various types of antibiotics, the expression level of pre-set housekeeping gene might be affected with varying degrees by different antibiotics. This means that the choice of housekeeping gene would be compromised according to the type of antibiotic selected, but there is still no literature available for researchers to refer to.

In this study, the reference strain *P. aeruginosa* PAO1 was set as the test strain, and the growth environment under the pressure of 8 antibiotics that could be used in molecular microbiology assays was simulated by adding 1/2 MIC of each antibiotic (Ducas-Mowchun et al., 2019). Then, a total of 10 candidate housekeeping genes were selected, and their expression levels were validated one by one by using quantitative real-time PCR. The aim of this study is to give some help to the initial step of reference gene selection in the studies of *P. aeroginosa*, and the experimental results show that it is necessary to select the housekeeping genes according to the different types of antibiotics used.

## Materials and methods

### Strains, resistance tests and culture conditions

*Pseudomonas aeruginosa* PAO1 strain was inoculated from a Lysogeny-Broth (LB) agar plates, and cultured overnight in LB liquid medium at 37°C in a rotary incubator shaker (200 rpm). Mid-exponential phase cultures were obtained after 1:100 dilution of overnight culture, and incubation at 37°C with shaking until OD_600_ ~ 0.5. Resistance profile of kanamycin, gentamycin, tetracycline, chloramphenicol, hygromycin B, apramycin, tellurite, and zeocin (Sangon) was determined by broth micro dilution assays as described by Clinical and Laboratory Standards Institute (2015). Experimental conditions by which strains were cultured under the pressure of antibiotics were simulated by adding 1/2 minimum inhibitory concentration (MIC) of corresponding antibiotics and incubation at 37°C with shaking overnight.

### Total RNA isolation and quantitative real-time PCR

Total RNA of antibiotic-induced strains was extracted from 5 ml mid-exponential phase cultures by using the Spin Column Bacteria Total RNA Purification Kit (Sangon) as described by the manufacturer. Genomic DNA was eliminated by RNase-free DNase Set (Sangon) treatment during the extraction procedure. Finally, total RNA was eluted from the column in a volume of 40 μl DEPC-treated water, and all RNA samples were uniformly diluted to ~25 μg/ml. This step is critical for performing successful calculations. The step of reverse transcription and real-time PCR were continuously carried out in the same reaction tube to effectively prevent contamination. Total RNA samples were transcribed to cDNA and amplified by using One Step TB Green Primescript Plus RT-PCR Kit (TaKaRa) according to the manufacturer’s protocol. Real-time PCR was carried out in the CFX96 ThermalCycler (Bio-Rad) under the reaction condition as 42°C at 5 min, 95°C at 10 s for cDNA synthesis, then followed by 40 cycles of 95°C for 5 s, 60°C for 20 s with data acquisition. At last, a melt curve analysis was performed using the temperature range 60°C to 95°C at 0.5°C intervals to confirm the specific amplification of a single PCR product. Primers were designed by using NCBI primer-Blast (Table 1). Cycle threshold (C_T_) values were determined with the Bio-Rad CFX Manager software and were used to calculate the gene expression level. All reactions were performed in triplicate, and the mean value was calculated.

**Table 1:** Primers used and characteristics of candidate housekeeping genes in this study.

Gene symbol	Forward primer (5′-3′)	Reverse primer (5′-3′)	Protein ID	Description
*algD*	TGTCGCGCTACTACATGCGTC	GTGTCGTGGCTGGTGATGAGA	AAG06928.1	GDP-mannose 6-dehydrogenase AlgD
*gyrA*	TGTGCTTTATGCCATGAGCGA	TCCACCGAACCGAAGTTGC	AAG03394.1	DNA gyrase subunit A
*anr*	CACCTTCCTGGTCAACCTGT	CTCGATGGAGTCGAGGATGT	AAG04933.1	Transcriptional regulator Anr
*nadB*	CTACCTGGACATCAGCCAC	GGTAATGTCGATGCCGAAGT	AAG04150.1	L-aspartate oxidas
*oprl*	ATGGAAATGCTGAAATTCGGC	CTTCTTCAGCTCGACGCGACG	AAG04362.1	Peptidoglycan associated lipoprotein
*recA*	TCCGCAGGTAGCACTCAGTTC	AAGCCGGATTCATAGGTGGTG	AAG07005.1	Recombinase A
*fabD*	GCATCCCTCGCATTCGTCT	GGCGCTCTTCAGGACCATT	AAG06356.1	Malonyl-CoA-[acyl-carrier-protein] transacylase
*proC*	CAGGCCGGGCAGTTGCTGTC	GGTCAGGCGCGAGGCTGTCT	AAG03782.1	Pyrroline-5-carboxylate reductase
*ampC*	AGATTCCCCTGCCTGTGC	GGCGGTGAAGGTCTTGCT	AAG07497.1	β-lactamase/D-alanine carboxypeptidase
*rpoS*	CTCCCCGGGCAACTCCAAAAG	CGATCATCCGCTTCCGACCAG	AAG07010.1	Sigma factor RpoS
*rpsL*	GCAAGCGCATGGTCGACAAGA	CGCTGTGCTCTTGCAGGTTGTGA	AAG07656.1	30S ribosomal protein S12

## Results

### MIC values of several antibiotics against *Pseudomonas aeruginosa* PAO1

The MIC values of kanamycin, tetracycline, chloramphenicol, gentamycin, apramycin, hygromycin B, zeocin, and tellurite were tested (Table 2), and the sub-MIC antibiotics were added during subsequent experiments to simulate the circumstances of molecular microbiology assays in which bacteria are cultured under the pressure of the above antibiotics.

**Table 2:** The minimum inhibitory concentrations (MICs) of the tested antibiotics against *Pseudomonas aeruginosa* PAO1.

Antibiotics	Kanamycin	Gentamycin	Tetracycline	Chloramphenicol	Hygromycin B	Apramycin	Tellurite	Zeocin
MIC (μg/ml)	16	1	4	16	32	4	2	16

The MICs were determined using the broth microdilution assay. Assays were performed in triplicates, and the mean value was calculated.

### Expression profiling of selected housekeeping genes

**Figure 1:**
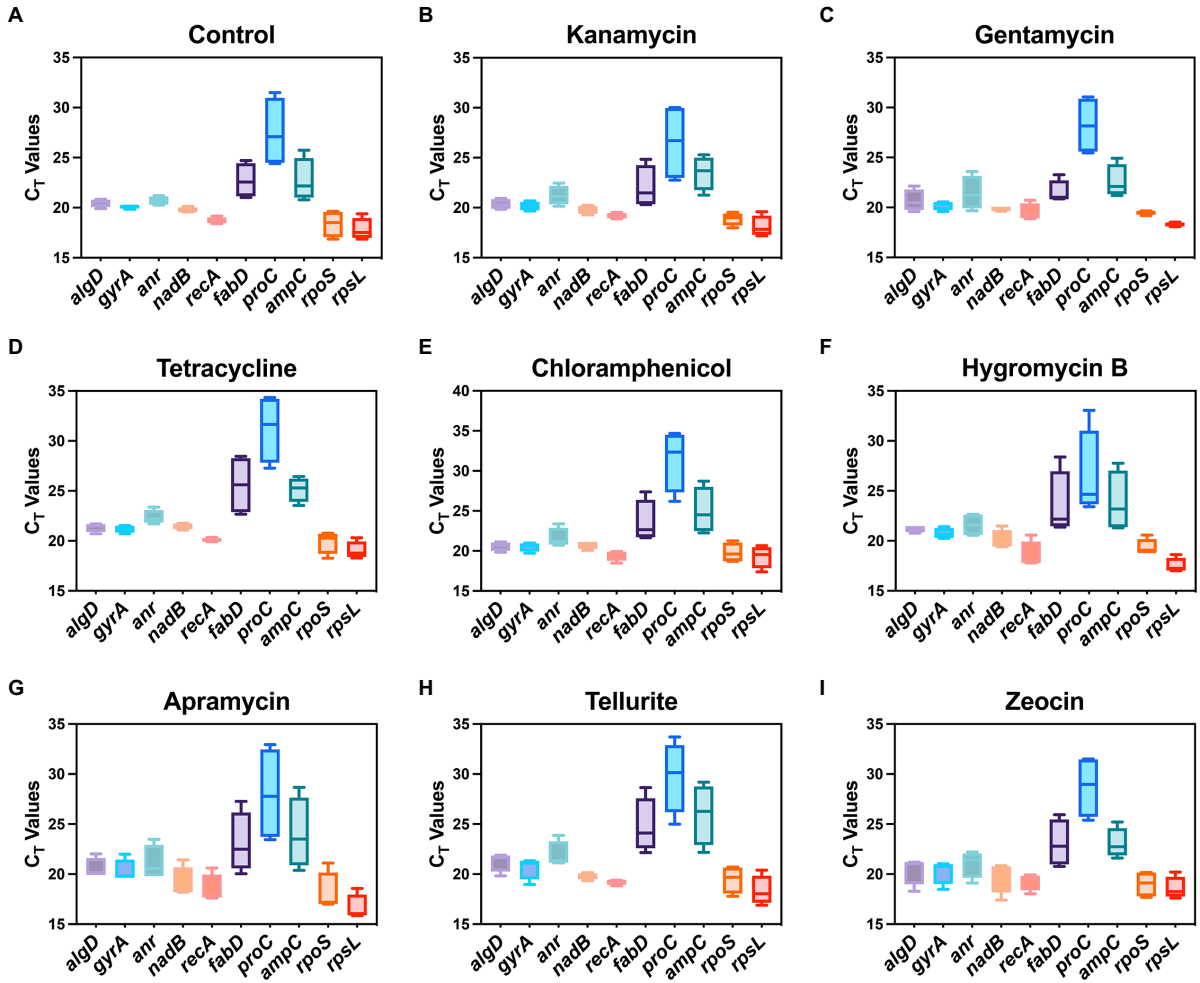
Range of real-time PCR cycle threshold (C_T_) values of 10 housekeeping genes in *P. aeruginosa* under 8 antibiotic pressures. **(A)** Control; **(B)** kanamycin; **(C)** gentamycin; **(D)** tetracycline; **(E)** chloramphenicol; **(F)** hygromycin B; **(G)** apramycin; **(H)** tellurite; **(I)** zeocin.

In this study, a total of ten housekeeping genes with different functions that have been used in other studies as internal controls of *P. aeruginosa* were selected (Table 1; Alqarni et al., 2016). The quantitative real-time PCR was then performed to validate the expression profile of candidate housekeeping genes by comparing the raw C_T_ values of each gene under the pressure of different antibiotics. Briefly, C_T_ values directly indicate the mRNA abundance of target genes, lower C_T_ value indicates higher abundance and vice versa. The C_T_ ranges of each tested gene under different antibiotic pressures are shown in Figure 1. C_T_ values of most candidate housekeeping genes such as *algD*, *gyrA*, *anr*, *nadB*, *recA*, *rpoS*, and *rpsL* basically appeared around 21 cycles, while *fabD* and *ampC* were slightly higher above average. The *proC* showed the highest C_T_ value around 28 cycles, indicating that its expression level is the lowest among all tested genes.

### Stability analysis of *Pseudomonas aeruginosa* housekeeping genes

To calculate the stability value of selected *P. aeruginosa* housekeeping gene in this study, dedicated algorithms including geNorm, NormFinder, BestKeeper and RefFinder were used, and an overall ranking is then carried out according to the comprehensive results.

#### geNorm algorithm

**Figure 2:**
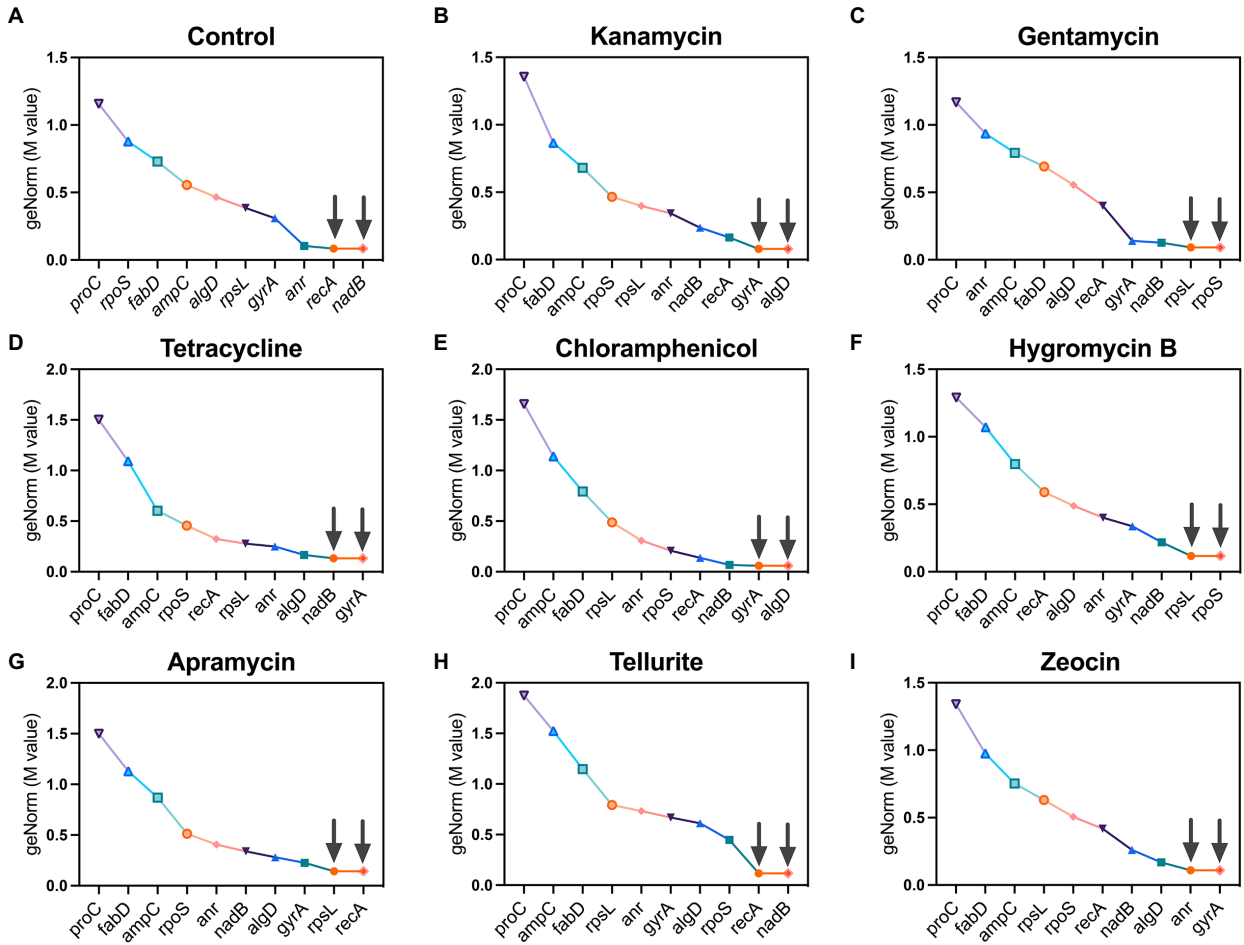
Ranking of 10 candidate housekeeping genes based on stability values (M values) calculated by geNorm algorithm. **(A)** Control; **(B)** kanamycin; **(C)** gentamycin; **(D)** tetracycline; **(E)** chloramphenicol; **(F)** hygromycin B; **(G)** apramycin; **(H)** tellurite; **(I)** zeocin.

geNorm is a classic algorithm used to determine the stability of candidate reference (housekeeping) genes by calculating a gene expression normalization factor (M value) based on the geometric mean of a user-defined number of reference genes. Genes with the lowest M value reflect the highest stability in terms of gene expression and vice versa, and each calculation provides two recommended housekeeping genes. Following this criterion, *recA* and *nadB* were found as suitable housekeeping genes in the control group which had no antibiotic pressure (Figure 2A). Under the pressure of kanamycin and chloramphenicol, *gyrA* and *algD* were found to be the most stable housekeeping genes (Figures 2B,E), while *rpoS* and *rpsL* registered the lowest M values under the pressure of gentamycin and hygromycin B (Figures 2C,F). In the group of tetracycline, *nadB* and *gyrA* were found to be the most stable housekeeping genes (Figure 2D), as well as the circumstance of *recA* and *rpsL* under the pressure of apramycin (Figure 2G). In the tellurite-treated samples, *recA* and *nadB* showed excellent stability (Figure 2H), while *anr* and *gyA* were found to be the most stable housekeeping genes under the zeocin pressure (Figure 2I).

#### NormFinder ranking

NormFinder was also used to screen the suitable candidate reference genes in this study. Similar to geNorm, this algorithm could rank the most competent housekeeping gene by calculating a stability value, with lower values representing more stability and vice versa. Under the rule of NormFinder algorithm, *anr* and *rpsL* exhibited excellent performance, and *anr* was the best housekeeping gene under the pressure of tetracycline, chloramphenicol, hygromycin B, apramycin and control group, while *rpsL* was the most stable gene in kanamycin, tellurite and zeocin group. Otherwise, *recA* was considered the best housekeeping gene only under the pressure of gentamycin (Table 3).

**Table 3:** Expression stability of seleceted *Pseudomonas aeruginosa* housekeeping genes calculated by NormFinder under different antibiotic stress.

Rank order	Control		Kanamycin		Gentamycin		Tetracycline		Chloramphenicol		Hygromycin		Apramycin		Tellurite		Zeocin	
	Gene	Index	Gene	Index	Gene	Index	Gene	Index	Gene	Index	Gene	Index	Gene	Index	Gene	Index	Gene	Index
1	*anr*	0.049	*rpsL*	0.181	*recA*	0.383	*anr*	0.122	*anr*	0.319	*anr*	0.227	*anr*	0.151	*rpsL*	0.217	*rpsL*	0.409
2	*nadB*	0.103	*algD*	0.353	*nadB*	0.453	*rpsL*	0.180	*rpsL*	0.756	*algD*	0.405	*rpoS*	0.313	*anr*	0.284	*gyrA*	0.449
3	*rpsL*	0.205	*gyrA*	0.473	*gyrA*	0.485	*algD*	0.438	*algD*	0.796	*gyrA*	0.542	*nadB*	0.438	*recA*	0.936	*anr*	0.472
4	*recA*	0.219	*anr*	0.496	*rpsL*	0.599	*ampC*	0.549	*gyrA*	0.859	*rpsL*	0.648	*recA*	0.761	*nadB*	1.016	*recA*	0.481
5	*ampC*	0.446	*recA*	0.706	*algD*	0.650	*nadB*	0.563	*nadB*	0.862	*nadB*	0.690	*rpsL*	0.801	*algD*	1.115	*algD*	0.634
6	*gyrA*	0.473	*nadB*	0.755	*rpoS*	0.669	*gyrA*	0.679	*fabD*	0.912	*rpoS*	0.714	*gyrA*	1.074	*fabD*	1.310	*nadB*	0.776
7	*algD*	0.747	*ampC*	0.920	*ampC*	0.885	*recA*	0.858	*recA*	0.960	*ampC*	1.030	*algD*	1.220	*gyrA*	1.358	*ampC*	0.987
8	*fabD*	1.047	*fabD*	0.966	*fabD*	1.056	*rpoS*	1.458	*rpoS*	1.135	*recA*	1.212	*ampC*	1.441	*rpoS*	1.640	*rpoS*	1.061
9	*rpoS*	1.572	*rpoS*	1.169	*anr*	1.201	*fabD*	2.385	*ampC*	1.832	*fabD*	1.675	*fabD*	1.551	*ampC*	2.392	*fabD*	1.303
10	*proC*	2.245	*proC*	3.290	*proC*	1.979	*proC*	3.093	*proC*	3.670	*proC*	2.093	*proC*	2.904	*proC*	3.200	*proC*	2.750

#### BestKeeper ranking

**Figure 3:**
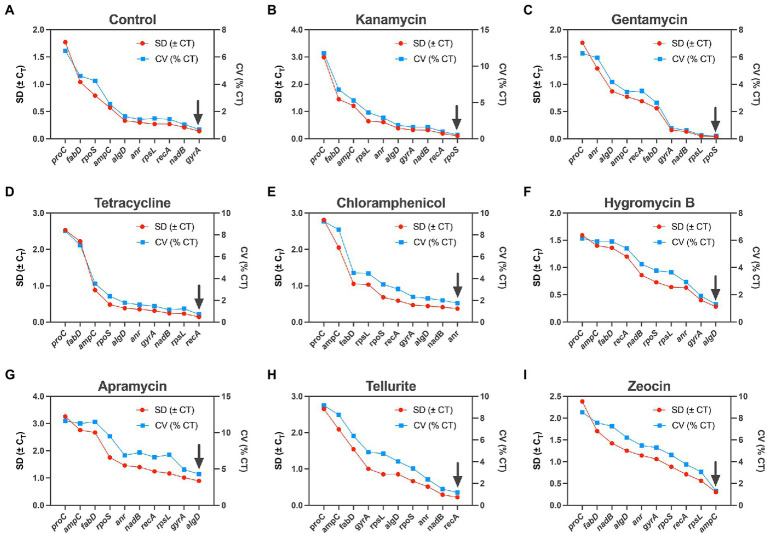
Expression analysis of 10 candidate housekeeping genes by BestKeeper algorithm. **(A)** Control; **(B)** kanamycin; **(C)** gentamycin; **(D)** tetracycline; **(E)** chloramphenicol; **(F)** hygromycin B; **(G)** apramycin; **(H)** tellurite; **(I)** zeocin.

BestKeeper is also a housekeeping gene ranking tool base on Microsoft Excel (Pfaffl et al., 2004). It could export a series of indices, of which standard deviation (SD) and coefficient of variance (CV) are two main parameters. Similarly, lower values represent higher level of stability, and only data with an SD < 1 is considered acceptable. The ranking of the housekeeping gene tested in our study by this algorithm was slightly different from the two methods mentioned above: *rpoS* was considered to be the most stable gene under the influence of kanamycin and gentamycin (Figures 3B,C), *recA* outperformed in tetracycline and tellurite (Figures 3D,H), *algD* was the most stable gene in apramycin and hygromycin B (Figures 3F,G), and *gyrA* and *anr* were considered the most stable gene in the control and chloramphenicol groups, respectively, (Figures 3A,E).

### Comprehensive ranking by RefFinder

**Figure 4:**
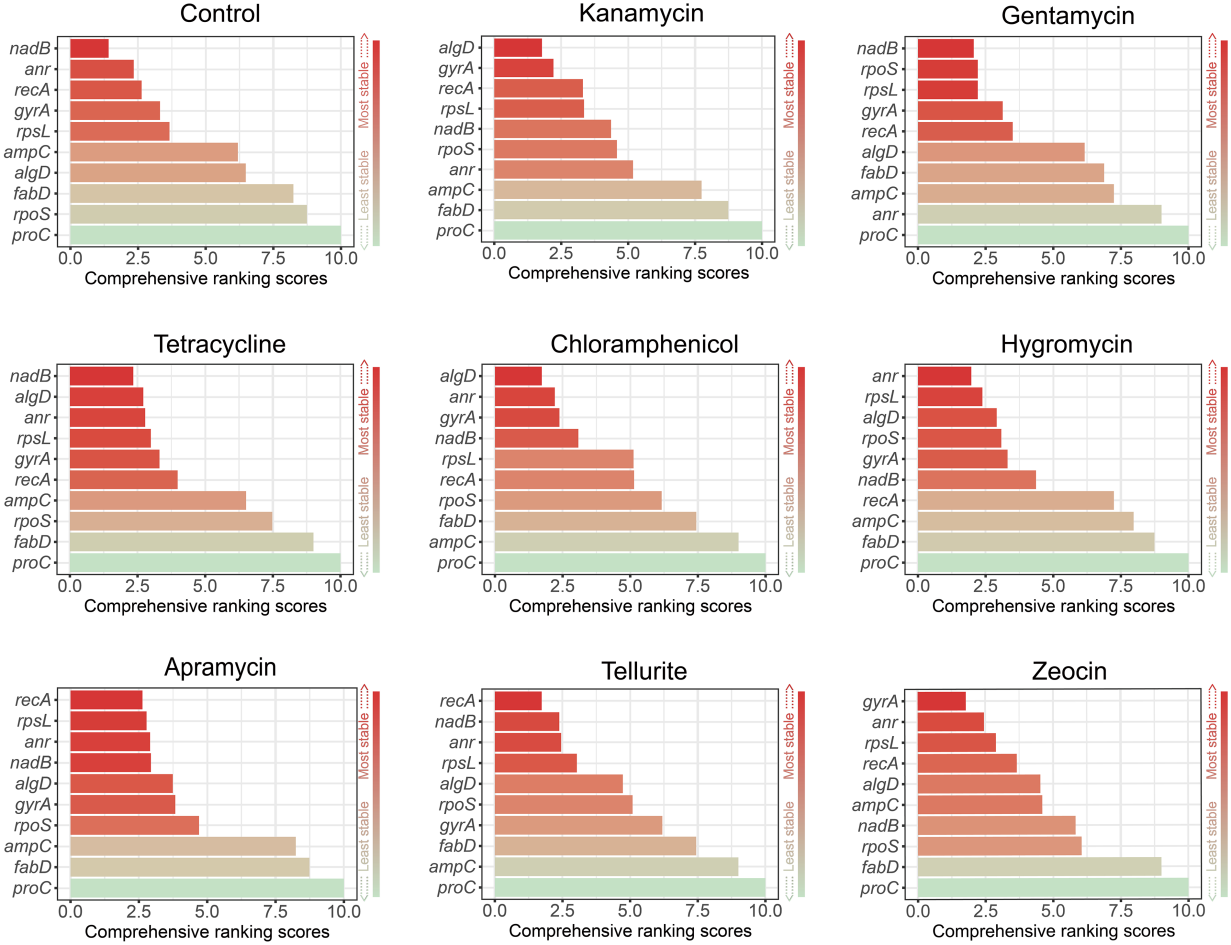
Comprehensive ranking by RefFinder according to the results of geNorm, NormFinder, and BestKeeper. Lower scores (red) indicate the higher expression stability.

RefFinder was used to generate a ranking order in light of the apparent ranking generated by geNorm, NormFinder, and BestKeeper (Xie et al., 2012). This algorithm assesses gene stability based on the above three algorithms plus ∆C_T_, and then generates a comprehensive ranking by ordering the geometric mean of multiple results. Similarly, the gene with the most stable expression level receives the lowest score. In general, as shown in Figure 4, *nadB* was the best housekeeping gene in the control group, gentamycin and tetracycline, *algD* performed best in kanamycin and chloramphenicol, *recA* got the lowest score in apramycin and tellurite group, and *anr* and *gyrA* were the best in hygromycin B and zeocin.

## Discussion

Over the past two decades, researchers have made great efforts to understand the natural characteristics of the human pathogen *P. aeruginosa*, and quantitative real-time PCR has proven indispensable in the study of this infamous, hard-to-eradicate pathogen. This technique has many advantages over other RNA quantification methods, such as high sensitivity, high reproducibility, and wide quantification range. At the same time, however, several variables must be carefully controlled to ensure the quality of the experimental results, such as the quality of the RNA sample extraction and reverse transcription process, a moderate amount of starting cDNA mass, an appropriate number of technical and biological replicates, and, of course, an appropriate selection of “housekeeping genes” as reference internal controls.

Ideally, this internal control gene should be highly conserved in the target species and held steady in ever-changing experimental conditions. However, it is not wise to attempt to chase a single all-around housekeeping gene as internal control, as no reference gene has been reported yet with verified stable expression under all circumstances, even for conserved genes involved in basic cellular processes throughout the life of bacteria. Several studies have shown that the expression levels of some commonly used housekeeping genes can fluctuate due to changes in the external environment, rendering them unsuitable as internal controls for experiments. Costaglioli et al. (2014) reported that the RNA expression levels of *P. aeruginosa* in 13 sputum specimens from nine cystic fibrosis patients showed that the commonly used housekeeping genes *clpX* and *oprL* were not as stable as *PA2875* and *PA3340* found by transcriptome sequencing, which are more suitable as housekeeping genes for clinical sample studies (Costaglioli et al., 2014). In another study, to evaluate the stability of *P. aeruginosa* housekeeping genes, Savli et al. (2003) collected 23 strains of *P. aeruginosa* with different resistance phenotypes and evaluated the stability of the six commonly used housekeeping genes and found that only *proC* and *rpoD* met the requirements for housekeeping genes, while *ampC*, *fabD*, *pbp-2* and *rpoS* were not recommended due to their poor stability (Savli et al., 2003). In addition, a study on *Pseudomonas brassicacearum* G20 showed that growth stage and growth environment should also be considered when selecting housekeeping genes. Bai et al. (2020) selected nine candidate housekeeping genes based on previous studies and tested the RNA expression level of the tested strain at different growth phases, temperatures, and environmental iron levels (Bai et al., 2020). After RNA extraction, quantitative real-time PCR and data analysis using BestKeeper, NormFinder and GeNorm algorithms, it was found that among the nine candidate housekeeping genes, only *rho, rpoD* and *gyrA* were suitable reference genes for *P. brassicacearum* GS20 (Bai et al., 2020).

The above study illustrates that the instability of housekeeping genes due to external environmental influences should be fully considered at the beginning of the experimental design, but this does not seem to have received enough attention, as many studies still select housekeeping genes solely based on the references they refer to. Therefore, it is very important to select one or several of suitable housekeeping gene in the initial stage of research, and it is for this reason that algorithms such as geNorm, NormFinder, BestKeeper, and RefFinder can be utilized, which can score and rank the stability of multiple candidate genes through programs to help us select the most suitable gene as internal control.

In most molecular microbiology assays, e.g., protein expression, gene deletion, bacterial two-hybrid system, transposon mutagenesis, etc., it is a common practice to introduce antibiotic resistance cassette carried by plasmid into the wild-type strains to be used as screening markers for subsequent experiments, resulting in the tested strains actually being cultured under the pressure of antibiotic throughout the experimental process (Sanchez-Romero et al., 1998; Ducas-Mowchun et al., 2019). Furthermore, since different types of antibiotics have different antimicrobial mechanisms, it is reasonable to assume that their effects on housekeeping gene stability are also heterogeneous. Therefore, it is valuable to clarify this issue so that we can select the appropriate housekeeping gene according to the type of antibiotics in the early stage of the experiment.

In this study, we selected 10 candidate housekeeping genes for *P. aeruginosa* PAO1 based on previous studies and determined their stability under the pressure of 8 antibiotics, among which kanamycin, gentamycin, tetracycline, and chloramphenicol are regular antibiotics widely used in most laboratories, while hygromycin B, apramycin, tellurite, and zeocin have rarely been used in the study of *P. aeruginosa*. These last four unconventional antibiotics are mainly used for cell culture or other purposes, but in the age of bacterial multidrug resistance, they have been already increasingly being used as select markers in the molecular microbiology studies (Blondelet-Rouault et al., 1997; Sanchez-Romero et al., 1998; Ducas-Mowchun et al., 2019). In general, the experimental results showed that different algorithms ranked the candidate housekeeping genes in different order, and no single reference gene was found to be competent for housekeeping gene under all antibiotic pressures (Figures 2, 3; Table 3). To draw a clear conclusion, RefFinder software was used to normalize the results of several algorithms, which showed that in the molecular microbiology assays, *algD* should be the first choice of housekeeping gene when we use kanamycin or chloramphenicol as screening antibiotics, and *recA* when we use apramycin or tellurite. On the other hand, when gentamycin or tetracycline are used as select agents, *nadB* is the best internal control gene, and *anr* and *gyrA* should be the first choice for hygromycin B and zeocin repectively (Figure 4). Actually, there is no need for us to pay too much attention to the actual ranking order of the best housekeeping genes, because several recent studies have reached a consensus that in the quantitative real-time PCR experiment, the selection of multiple housekeeping genes as internal control instead of one is required. Therefore, our work can be used in the initial stage of the experiment to guide the selection of internal controls based on the type of antibiotic used, and two or more genes can be easily selected as internal controls according to the comprehensive ranking of housekeeping genes shown in Figure 4. It is worth noting that there may be other excellent housekeeping genes that have not yet been discovered. In future work, transcriptome sequencing can be used to screen for genes that are stably expressed under different conditions and make them “candidate housekeeping genes,” and further validate their stability using qRT-PCR. This study provides a comprehensive summary of the effects of commonly used laboratory antibiotics on the stability of housekeeping genes in *P. aeruginosa* to provide guidance and suggestions for housekeeping gene selection in future studies.

## Data availability statement

The original contributions presented in the study are included in the article/supplementary material, further inquiries can be directed to the corresponding authors.

## Author contributions

ChaL, HS, and XC designed the project and drafted the manuscript. LM and ChuL conducted the experiments. JL performed the statistical analysis. HX, JS, and MH helped with the writing. All authors contributed to the article and approved the submitted version.

## Funding

This work was supported by the National Natural Science Foundation of China (81902124 and 82002205).

## Conflict of interest

The authors declare that the research was conducted in the absence of any commercial or financial relationships that could be construed as a potential conflict of interest.

## Publisher’s note

All claims expressed in this article are solely those of the authors and do not necessarily represent those of their affiliated organizations, or those of the publisher, the editors and the reviewers. Any product that may be evaluated in this article, or claim that may be made by its manufacturer, is not guaranteed or endorsed by the publisher.
